# Functional Disconnection of the Angular Gyrus Related to Cognitive Impairment in Patients With Type 2 Diabetes Mellitus

**DOI:** 10.3389/fnhum.2021.621080

**Published:** 2021-02-03

**Authors:** Fei Qi, Dongsheng Zhang, Jie Gao, Min Tang, Man Wang, Yu Su, Yumeng Lei, Zhirong Shao, Xiaoling Zhang

**Affiliations:** ^1^Department of MRI, Shaanxi Provincial People’s Hospital, Xi’an, China; ^2^Department of Graduate, Xi’an Medical University, Xi’an, China

**Keywords:** type 2 diabetes mellitus, resting-state fMRI, functional connectivity, angular gyrus, neuroimaging

## Abstract

Type 2 diabetes mellitus (T2DM) is related to a variety of cognitive impairments that may even progress to dementia. Studies have found the angular gyrus (AG) is a cross-modal integration hub that is involved in a variety of cognitive processes. However, few studies have focused on the patterns of resting-state functional connections (rsFCs) of the AG in patients with T2DM. This study explored the functional connection (FC) between the AG and the whole brain and the relationship between the FC and clinical/cognitive variables in patients with T2DM. 44 patients with T2DM and 43 sex-, age-, and education-matched healthy controls underwent resting-state fMRI and received neuropsychological assessments. Compared with the control group, the T2DM group showed abnormal rsFCs between the AG and multiple brain regions. The FC between the left AG and the left medial temporal lobe in the T2DM group was positively correlated with scores on the Montreal Cognitive Assessment, after a Bonferroni correction (*r* = 0.40, *P* = 0.009). Collectively, patients with T2DM have abnormal FCs between the AG and extensive brain regions that may be related to various cognitive processes.

## Introduction

Type 2 diabetes mellitus (T2DM) is a heterogeneous metabolic disorder characterized by reduced insulin sensitivity and relative insulin deficiency, which increases the risk for cognitive decline and dementia, such as Alzheimer’s disease (AD) and vascular dementia (Biessels et al., 2006; Kopf and Frolich, 2009; McCrimmon et al., 2012). A large number of studies have found that various cognitive functions, such as memory, attention, executive function, language comprehension, and problem solving processes, in patients with T2DM have varying degrees of impairment (Ruis et al., 2009; Pasquier, 2010). However, the potential neuropathological mechanism of cognitive impairment in T2DM is currently unclear.

The human cerebral cortex consists of many neurons that are organized into a complex network that forms the structural substrate for cognitive functioning (Hagmann et al., 2008). Resting-state fMRI is a powerful tool that can effectively enable investigating the neural activity of the human brain and this methedhas been widely used to examine the pathogenesis of various neuropsychiatric disorders (Biswal et al., 1995; Zhang and Raichle, 2010). Resting-state functional connectivity (rsFC) is a commonly used method to evaluate inter-regional functional coupling between different brain regions. Research that has used the rsFC technique to study patients with T2DM has identified disrupted rsFCs in several key brain regions that are involved in cognitive processing, such as the posterior cingulate gyrus (Tan et al., 2019), hippocampus (Zhou et al., 2010), thalamus (Chen et al., 2015), and the amygdala (Xia et al., 2018). These brain areas are associated with abnormal congitive changes including execution, memory, and emotions. In addition to these regions, the angular gyrus (AG) has recently been found to be involved in a variety of cognitive processes (Seghier, 2013), and it appears to be a functional convergence zone (Ramanan et al., 2018). Especially in human episodic memory retrieval and semantic processing, AG is one of the most consistently implicated brain regions (Binder et al., 2009; Ramanan et al., 2018). Studies have confirmed that damage to the AG can produce a variety of cognitive dysfunctions, such as aphasia, finger agnosia, spatial disorientation, acalculia, and agraphia (Corbett et al., 2009; Ardila, 2014).

Large-scale connectivity analyses have shown that the AG is one of the major connector hubs that connect different subsystems (Hagmann et al., 2008; Tomasi and Volkow, 2011). Anatomically, the AG is located behind the inferior parietal lobule junction between the occipital, temporal, and parietal lobes (Seghier, 2013). It is considered an important interface for conveying and integrating information (Tomasi and Volkow, 2011). Its rich pattern of anatomical connectivity enables considerable interactivity between the AG and temporofrontal subsystems, in addition to other medial regions, such as the hippocampus, caudate, and precuneus (Seghier, 2013). Functionally, the AG is deeply involved in multiple tasks and processes, such as semantics (Binder et al., 2009; Binder and Desai, 2011), memory (Vilberg and Rugg, 2008; Ramanan et al., 2018), attention (Singh-Curry and Husain, 2009), and emotional control (Kohn et al., 2014). Specifically, given its rich connectivity and its similarity to a functional gathering center, the AG resembles a cross-modal integrative hub where converging multisensory information is combined and integrated to comprehend and make sense of events, reorient attention, and solve familiar problems, which play an important role in a variety of cognitive processes (Seghier, 2013).

Multiple neuroimaging studies have confirmed the abnormal structure and function of the AG in patients with T2DM. For example, the cortical thickness of the AG was found to be reduced in patients with T2DM (Peng et al., 2015). In addition, patients with T2DM exhibit significantly lower cerebral blood flow (Bangen et al., 2018), decreased spontaneous neuronal activity (Wang et al., 2017), and several studies have found aberrant AG rsFCs (Liu et al., 2018; Tan et al., 2019). Liu et al. (2016) demonstrated that aberrant functional connectivity anchoring in the AG might serve as a neuroimaging marker for T2DM-related cognitive decline. A PET study also found that the reduction in regional cerebral glucose metabolic rate in the posterior cingulate cortex, precuneus, the anterior and inferior prefrontal cortices and AG was associated with an increased risk of AD in patients with pre-diabetes and T2DM (Baker et al., 2011). These findings raise the possibility that the AG may be a vulnerable brain region in patients with T2DM. Thus, it is extremely important to ascertain the intrinsic pattern of rsFCs of the AG in patients with T2DM.

Considering the important role of the AG in cognitive function, revealing the intrinsic pattern of rsFCs of the AG should be helpful for clarifying the neuropathological mechanism of cognitive impairment in T2DM. Therefore, we aimed to investigate the intrinsic rsFCs of the AG in patients with T2DM using rs-fMRI, and to explore the association between abnormal AG connections and patients’ cognitive performance. We hypothesized that there is a wide range of functional connectivity abnormalities between the AG and the whole brain in T2DM and that these abnormalities are correlated with cognitive scores.

## Materials and Methods

### Participants

A total of 50 patients with T2DM and 45 gender-, age-, and education-matched healthy controls (HCs) were recruited for our study from March 2018 to December 2019 at Shaanxi Provincial People’s Hospital. All the participants received a detailed medical history interview and a neurological examination. Clinical and demographic information were collected for all subjects, including biological tests, blood pressure, body mass index (BMI), educational level, and duration of the disease (only for patients with T2DM). Blood samples were obtained after overnight fasting for at least 8 h to test the levels of fasting blood glucose. The inclusion criteria were as follows: (1) all participants were 40–70 years old, right-handed, and received at least six years of education; (2) the diagnosis of T2DM was based on the 2014 American Diabetes Association criteria, and stable therapy (diet, oral medications, and/or insulin), fasting plasma glucose (FPG) levels ≥7.0 mmol/L or 2 h OGTT glucose levels ≥11.1 mmol/L; and (3) all the HCs in the study had to have normal fasting glucose levels and FPG levels ≤6.1 mmol/L. The exclusion criteria were as follows: (1) a history of hypoglycemic episodes or macrovascular diseases (e.g., cerebrovascular or cardiovascular diseases), or hypoglycemia (blood glucose <3.9 mmol/L), or hyperglycemia (blood glucose >33.3 mmol/L) during hospitalization; (2) a history of brain lesions, such as cerebrovascular accidents, tumors, traumas, infections, or other diseases; (3) a stroke, or alcohol or other substance dependence; (4) an endocrine disease, such as thyroid dysfunction; (5) Parkinson’s disease, major depression or other disorders that could affect cognitive function; (6) an MMSE score <24 (for HCs only); or (7) MRI contra-indications.

All the patients were instructed to control their blood glucose according to their doctor’s orders on the day of the scan. They arrived at the Department of MRI between 6:00 and 7:00 pm after dinner, and a structured clinical interview and neuropsychological tests were conducted for approximately 30 min. Then, the patients were prepared for the MRI scan. Only one patient was scheduled each day to ensure that each patient completed the MRI scan within 2 h after dinner. The HCs’ test procedures and scan times were comparable to those of the patients. The study was approved by the Ethics Committee of Shaanxi Provincial People’s Hospital. All the subjects were informed in detail of the trial content and methods and signed an informed consent form before the examination. Details of the complications and therapeutic agents for T2DM are provided in Supplementary Tables 1, 2.

### Neuropsychological Tests

All the participants completed standardized neuropsychological tests to assess their mental state and cognitive functioning. The Mini-Mental State Examination (MMSE) and the Montreal Cognitive Assessment (MoCA) were used to assess general cognitive function. Information processing speed and attention were tested by the Trail Making Test A (TMT-A). Executive function and visuospatial skills were evaluated by the Clock Drawing Test, and the Beck Depression Inventory was used to evaluate depressive symptoms. The tests were conducted by psychologists trained in systematic testing.

### Image Acquisition

All the MRI images were acquired using the 3.0-Tesla MR scanner (Philips Ingenia, Best, Netherlands) with an 16-channel phased-array head coil. First, routine T1WI, T2WI, and T2-FLAIR scans were acquired to exclude visible brain lesions. An age-related white matter change scale was used to quantitatively evaluate lacunar infarcts and white matter hyperintensity based on fluid attenuated inversion recovery images; subjects with a score >2 were excluded. Resting-state functional BOLD images were obtained using a gradient-echo planar sequence with the following parameters: TR = 2,000 ms, TE = 30 ms, slices = 34, thickness = 4 mm, gap = 0 mm, FOV = 230 mm × 230 mm, matrix = 128 × 128, FA = 90°, and 200 volumes. Sagittal 3-dimensional T1-weighted images were acquired with the following parameters: TR = 7.5 ms, TE = 3.5 ms, FA = 8°, FOV = 250 mm × 250 mm, matrix = 256 × 256, slice thickness = 1 mm, no gap, and 328 sagittal slices. All the participants were instructed to keep their eyes closed but stay awake during the scan. Head motion and scanner noise were reduced using foam pads and headphones.

Data from eight subjects (six patients with T2DM and two HCs) were excluded due to head motion and white matter hyperintensity problems. Finally, a total of 44 patients with T2DM and 43 HCs were enrolled in the study.

### Data Preprocessing

Data preprocessing was performed using the programs in Data Processing and Analysis for Brain Imaging v3.0^1^ based on Statistical Parametric Mapping v12 (SPM12).^2^ The slice timing and realignment to correct for head motion were performed after discarding the first 10 time points. Any head motion >1.5 mm or translation >1.5° rotation in any direction was excluded. To normalize the fMRI data, the functional images were co-registered to high-resolution T1WI images, and the resulting aligned T1 dataset was transformed into Montreal Neurological Institute (MNI) space (re-sampling voxel size = 3 mm × 3 mm × 3 mm). The obtained images were smoothened with a 6 mm full-width half-maximum isotropic Gaussian kernel. In order to decrease spatial bias and the impact of high-frequency noise, detrending and filtering (0.01–0.08 Hz) were applied. Multiple regression models were used to remove the effects of covariates of no interest, which involved 24 motion parameters, cerebrospinal fluid signals, and white matter signals.

### Functional Connectivity Analysis of AG

We chose the left and right AG as seed regions, defined in standard MNI space, using the map of the Automated Anatomical Labeling Atlas. Correlations were computed between the mean signal change and the time series of every voxel of the whole brain for each ROI. The resulting correlation coefficients (*r*) were converted by the Fisher’s *r*-to-*z* transformation to improve the Gaussianity of their distribution. The ROIs and intergroup analysis results were visualized using the BrainNet Viewer package.^3^

### Statistical Analysis

SPSS 18.0 was used to conduct the statistical analysis. Independent sample *t*-tests (two-tailed) were used to compare group differences for normally distributed variables. Variables that were not normally distributed were analyzed using the Mann–Whitney U-test. The chi-square (χ^2^) test was used to assess intergroup differences in gender. The significance level was set at *P* < 0.05.

Voxel-wise two-sample *t*-tests embedded in Data Processing and Analysis for Brain Imaging were performed to evaluate group differences in resting-state FC for each ROI. The significance was determined using a Gaussian random field correction with *P* < 0.05 (voxel *P* < 0.001, cluster size >52).

The mean FC values of the functionally altered brain areas between the groups were extracted from the patients with T2DM. Correlations were performed to identify the relationships between the mean FC and clinical/cognitive variables.

## Results

### Clinical and Neuropsychological Data

Table 1 shows the demographic, clinical, and cognitive data. There were no significant group differences in age, gender, educational level, BMI, total cholesterol, triglycerides, low-density lipoprotein, or blood pressure (*P* > 0.05). However, the T2DM group had increased levels of fasting blood glucose, glycated hemoglobin, and Beck Depression Inventory scores than the HC group did (all *P-*values < 0.05). The T2DM group exhibited poorer performance on the MoCA (*P* < 0.05). The MoCA subtest scores are provided in Supplementary Table 3.

**TABLE 1:** Demographic, clinical, and cognitive data of the patients with type 2 diabetes mellitus (T2DM) and the healthy controls (HCs).

Variable	T2DM (*n* = 44)	Controls (*n* = 43)	*P*-value
Age (years)	56.14 ± 5.73	54.37 ± 4.90	0.13
Male/female	30/14	28/15	0.71^#^
Educational level (years)	14.05 ± 1.84	14.79 ± 2.39	0.11
Diabetes duration (years)	10.93 ± 6.34	–	–
Systolic BP (mmHg)	124.84 ± 16.77	122.33 ± 7.81	0.37
Diastolic BP (mmHg)	78.54 ± 9.93	81.63 ± 5.43	0.08
BMI (kg/m^2^)	23.95 ± 2.66	24.14 ± 3.13	0.77
FBG (mmol/L)	8.78 ± 2.97	5.23 ± 0.81	<0.01
HbA1c (%)	8.16 ± 1.99	5.56 ± 0.54	<0.01
TG (mmol/L)	1.47 ± 0.64	1.80 ± 1.21	0.12
TC (mmol/L)	4.51 ± 1.02	4.83 ± 0.91	0.14
LDL (mmol/L)	2.49 ± 0.77	2.82 ± 0.90	0.07
MMSE	27.72 ± 2.16	28.35 ± 1.65	0.14
MoCA	25.16 ± 2.87	27.00 ± 1.73	<0.01
TMT-A	78.98 ± 22.83	68.73 ± 27.06	0.06
CDT	18.72 ± 8.61	21.07 ± 6.51	0.16
BDI	0 (0,16)	0 (0,5)	<<0.01^Δ^

*Normally distributed variables are presented as mean ± standard deviation and variables that are not normally distributed are presented as median (minimum, maximum). BMI, body mass index; FBG, fasting blood glucose; TG, triglycerides; TC, total cholesterol; LDL, low-density lipoprotein; HbA1c, glycated hemoglobin; MMSE, Mini-Mental State Examination; MoCA, Montreal Cognitive Assessment; TMT-A, Trail Making Test A; CDT, Clock Drawing Test; BDI, Beck Depression Inventory. ^Δ^*P* for the Mann-Whitney *U*-test. ^#^*P* for the χ^2^ test.*

### The FC Between the AG and the Cerebral Cortex

**FIGURE 1:**
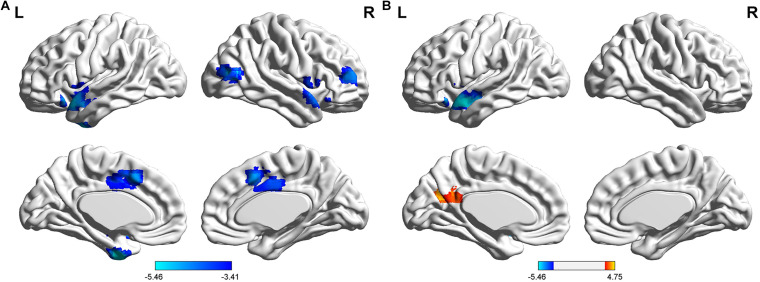
Differences in resting-state functional connectivity between the T2DM group and the HC group (two-sample *t*-test, *P* < 0.05, Gaussian random field-corrected). **(A)** Lower connectivity of the left AG in the T2DM group. **(B)** Lower connectivity of the right AG in the T2DM group.

The FCs between the left AG and the left medial temporal lobe (MTL), bilateral temporal Pole, right middle frontal gyrus (MFG), right middle temporal/occipital gyrus, and bilateral middle cingulate gyrus (MCC) were significantly lower in the T2DM group than in the HC group (*P* < 0.05). Compared to the HC group, the T2DM group had decreased FC between the right AG and left the temporal Pole. By comparison, the T2DM group had increased FC between the right AG and the left cuneus/precuneus (*P* < 0.05, Figure 1 and Table 2).

**TABLE 2:** Abnormal functional connectivity in the patients with type 2 diabetes mellitus compared to the healthy controls.

Seed ROI	Brain region	Peak MNI coordinates			Voxel (mm^3^)	BA	*t*-value
		X	Y	Z			
L angular gyrus	L meidal temporal lobes	−27	−3	−36	65	20/36	−5.73
	L temporal pole	−48	−15	−15	119	38/22	−5.11
	R temporal pole	63	12	0	86	38/22	−4.67
	R middle frontal gyrus	36	48	12	52	10/46	−4.50
	R middle temporal/occipatal gyrus	39	−69	12	60	37	−5.12
	B middle cingulate gyrus	0	15	42	131	32/24	−4.67
R angular gyrus	L temporal pole	−51	6	−15	117	38/22	−5.46
	L cuneus/precuneus	−15	−57	24	66	31	4.75

*BA, Brodmann’s area; MNI, Montreal Neurological Institute; B, bilateral; L, left; R, right. Group differences in functional connectivity were evaluated by two-sample *t*-tests (*P* < 0.05, Gaussian random field-corrected).*

### Correlation Between the FC and Clinical/Cognitive Variables

**FIGURE 2:**
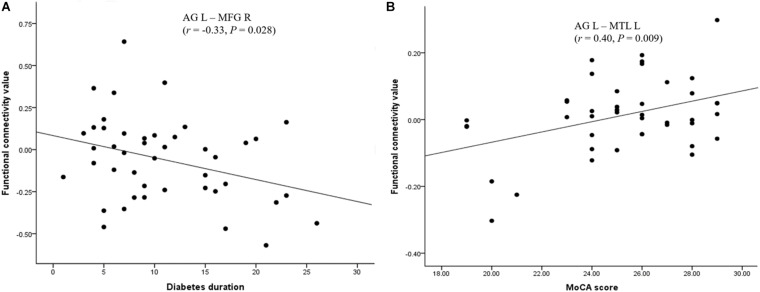
Significant correlation between the FC of the AG and neuropsychological performances in the T2DM group. **(A)** Significant positive correlation of MoCA scores with the FC between the left AG and the left MTL. **(B)** Significant negative correlation of diabetes duration with the FC between the left AG and the right MFG. AG: angular gyrus; MTL: medial temporal lobe; MFG: middle frontal gyrus; MoCA: Montreal Cognitive Assessment.

The FC between the left AG and the left medial temporal lobe was positively correlated with the MoCA score (*r* = 0.40, *P* = 0.009), after a Bonferroni correction. The FC between the left AG and the right middle frontal gyrus was negatively correlated with diabetes duration (*r* = −0.33, *P* = 0.028); however, this correlation was not significant after applying the Bonferroni correction (Figure 2). There was no significant correlation between clinical/cognitive variables and FC values in HC group.

## Discussion

This study explored the patterns of rsFCs of the AG in patients with T2DM for the first time. We observed that the bilateral AG exhibited abnormal FCs with multiple brain regions in patients with T2DM compared to the HCs, and the reduced FC between the left AG and the left medial temporal lobe was positively correlated with the MoCA. These results suggest the AG is a cross-modal integration hub, and its extensive functional disconnectivity may be involved in the neuropathology of cognitive impairment in patients with T2DM.

The AG and the MTL are the key regions of the core memory network, especially for episodic memory (Rugg and Vilberg, 2013). The MTL provides the core elements of episodic representation (i.e., “who,” “what,” “when,” and “where”) and the multimodal integration of contextual details is modulated by the AG (Bonnici et al., 2016). Vivid event representation is realized through MTL-AG interactions, whereby the MTL curates and flexibly binds the detailed contextual layer onto the crux representation to create a perceptually rich and spatially coherent memory representation (Ramanan et al., 2018). The decreased FC observed in the current study between the left AG and left MTL of patients with T2DM may indicate that the interaction between the MTL and AG is disordered in such as way that the core factors and contextual information in episodic memory cannot be reasonably integrated and lead to episodic memory impairment in patients. Studies have found that the impairment of episodic memory in patients with T2DM is related to MTL abnormalities (Arvanitakis et al., 2004; Rolandsson et al., 2008; Zhou et al., 2010), whereas our results suggest that the impairment of episodic memory is caused by abnormal information integration. Our study provides a new perspective for revealing the neuromechanism of episodic memory impairment in patients with T2DM.

Type 2 diabetes mellitus is a strong risk factor for AD and it has a neuropathological mechanism similar to AD (Jones et al., 2009). Research by Zheng et al. suggest that disrupted functional connectivity in the MTL may reflect an early pathological change in the development of AD, and it might be one of the earliest preclinical changes in AD (Song et al., 2015). Similarly, impairment of MTL-mediated episodic memory has been reported in patients with impaired glucose tolerance and diabetes (Rolandsson et al., 2008). The typical manifestations of AD are early progressive impairment of episodic memory, followed by a variety of cognitive dysfunctions, such as language and visual perception dysfunctions, and subsequent emotional and behavioral abnormalities (Silva et al., 2019). We found that the FC values between the left AG and the left MTL were positively correlated with MoCA scores, which may indicate that the abnormal episodic memory caused by the interruption of functional connectivity may further affect the overall cognitive functioning of patients. Thus, we speculate that the functional disconnection between the AG and the MTL also plays an important role in overall cognitive impairment in T2DM.

Semantic processing is supported by multiple brain areas, including the dorsal and ventral posterior frontal lobe, the posterior middle temporal cortex, and the AG (Noonan et al., 2013). The anterior temporal lobe (ATL) mainly acquires semantic representations (Patterson, 2007; Pobric et al., 2010), while the frontal and posterior middle temporal gyrus and the AG constitute a network with semantic control (Lambon Ralph et al., 2009; Pobric et al., 2010; Whitney et al., 2011; Robson et al., 2012; Noonan et al., 2013), which can allocate attention to integrate information sources according to requirements and combine external information with internal representations. Based on prior evidence, functional coupling between the AG and the ATL may reflect engagement in more detailed semantic processes in an integrative attempt to form new abstract meanings (Molinaro et al., 2015). Our results show that the reduced FC between the AG and the temporal Pole might indicate an abnormality in the semantic processing of patients with T2DM. In addition, we found that patients with T2DM performed poorly in the naming, verbal retelling, and abstract ability subtests of the MoCA, which also confirms our speculation. Besides the temporal pole, we also observed disrupted connectivity between the AG and the middle frontal gyrus and temporal–occipital junction, which may be because all the subjects in this study were Chinese. Although the overall networks for Chinese character-processing are congruent with the alphabetic language networks (Bolger et al., 2005; Tan et al., 2005; Wu et al., 2012), there is increasing evidence that Chinese characters may be processed differently from alphabetic languages due to the unique linguistic features of the characters. Chiao et al. noted that the middle frontal gyrus and the temporal–occipital junction participated in more complex semantic processing and transformation when processing Chinese characters, due to the unique language characteristics of the characters (Wu et al., 2012). Therefore, we speculate that a disorder of the semantic control network in T2DM may be one of the reasons for the impairment of semantic cognitive functioning in patients with T2DM. Studies have confirmed that the decreased neuronal activity in the MTG (van Schie et al., 2005; Peng et al., 2016) and decreased integrity of white matter fiber bundles involved in language and semantic processing are related to abnormal semantic cognitive function (Chang et al., 2015; Tan et al., 2016). Our findings supplement the evidence on the neural mechanism of semantic cognitive impairment in T2DM from the perspective of the functional disconnection of the AG. The correlation results showed that the FC values between the left AG and the right MFG were negatively associated with disease duration, which may indicate that the impairment of semantic cognitive functioning will gradually increase with the length of the disease’s course. However, this hypothesis may only apply to patients with T2DM whose mother tongue is Chinese.

Anatomically, the AG has projections to the MCC (Vogt, 2005) that are functionally involved in cognitive emotion regulation (Kohn et al., 2014). Mel’nikov et al. (2018) showed that the activity of the MCC and the left parietal lobule are reduced by negative emotional stimuli, which may be considered a potential diagnostic marker for depressive disorders and a target for neurofeedback. T2DM is known to be associated with an increased risk of depression (Musselman et al., 2003), and some studies suggest that the relationship between depression and T2DM is bi-directional or co-morbid (O’Connor et al., 2009). The depression scores of patients with T2DM were significantly higher than those of the healthy participants in our study; therefore, the decreased FC between the left AG and the bilateral MCC may be related to an emotional abnormality in patients with T2DM.

The precuneus and AG are the core regions of the Default Mode Network (DMN), which is extensively involved in integrating information (Cavanna and Trimble, 2006), and plays an important role in various cognitive functions, such as episodic memory retrieval and emotion mediation. Research has confirmed that the nodes within the network are inherently interdependent and highly complementary in function. The reduction of functional connectivity in damaged local brain areas requires compensatory increases in other functional areas. These nodes are interdependent and maintain a dynamic balance (Andrews-Hanna et al., 2010). We found the FC between the left cuneus/precuneus and the right AG was increased, which may be a compensatory mechanism to counteract the effects of cognitive deficits and maintain normal cognitive functioning.

Interestingly, this study used the left and right AG as ROIs, respectively. We found that, compared with the right AG, there was a wider disconnection between the left AG and the whole brain, which may be due to the laterality of the AG in episodic memory and semantic processing. Studies have reported that the left AG seems to be engaged in all aspects of semantic processing that require concept retrieval and conceptual integration (Binder et al., 2009), and it is one of the most consistently implicated brain regions in the retrieval of episodic memory (Ramanan et al., 2018). This may be one of the reasons for the laterality of the results of this study.

### Limitations

This study has some limitations. First, structural changes have a potential impact on function, and the AG has rich anatomical connections. Structural and functional connections should be combined in future studies to explore their role in cognitive impairment in T2DM. Second, the medications of patients with T2DM were not identical, so the study’s results may be biased due to the confounding effect of drugs. Third, score of the MMSE test in two diabetic patients were less than 24. Although the results of this study did not change after excluding these two subjects, this may still produce a certain bias in the results Fourth, although the MoCA score is composed of multiple tests, such as attention, language repetition, abstract ability, and delayed memory, its evaluation of performance is insufficient. We will add some comprehensive and effective cognitive test in the future, so we can further explore the relationship between AG dysfunction and the impairment of different cognitive functions in patients with T2DM. Finally, we will take AG and other hubs into combinative analysis, which will contribute to in-depth discussion of the interaction between these huds and the relationship between the network formed by these hubs and cognitive function.

## Conclusion

In summary, this study is the first to reveal the rsFC pattern of the AG in patients with T2DM, which indicates the AG has abnormal functional connections with extensive brain regions that may affect various cognitive processes. These findings suggest that the disconnection of the AG plays an important role in the cognitive impairment of patients with T2DM, which provides a new perspective to reveal the neuropathological mechanism of the cognitive impairment of T2DM.

## Data Availability Statement

The raw data supporting the conclusions of this article will be made available by the authors, without undue reservation.

## Ethics Statement

The study was approved by the Ethics Committee of Shaanxi Provincial People’s Hospital. All the subjects were informed in detail of the trial content and methods and signed an informed consent form before the examination.

## Author Contributions

FQ and DZ drafted the manuscript, designed the experiment, and performed the statistical analysis. JG contributed to performing the experiments and revised the manuscript. MT, MW, and YS collected the data. YL and ZS provided the technical support. XZ made contributions to the design of the experiment and revised the manuscript. All authors read and approved the final manuscript.

## Conflict of Interest

The authors declare that the research was conducted in the absence of any commercial or financial relationships that could be construed as a potential conflict of interest.

## Footnotes

1 http://rfmri.org/dpabi

2 http://www.fil.ion.ucl.ac.uk/spm

3 http://www.nitrc.org/projects/bnv/
