# Supplementary material for: Functional Disconnection of the Angular Gyrus Related to Cognitive Impairment in Patients With Type 2 Diabetes Mellitus
Source: Front Hum Neurosci. 2021 Feb 3;15:621080. doi: 10.3389/fnhum.2021.621080 (PMC7886792; doi:10.3389/fnhum.2021.621080)
Supplement: Supplementary file 1 [file Table_1.docx]

**Supplementary Table 1:** T2DM complications

| Complication | Number of patients |
| --- | --- |
| No complications | 28 |
| Nephropathy | 4 |
| Peripheral neuropathy | 4 |
| Retinopathy | 3 |
| Nephropathy + peripheral neuropathy | 5 |
