# Supplementary material for: Functional Disconnection of the Angular Gyrus Related to Cognitive Impairment in Patients With Type 2 Diabetes Mellitus
Source: Front Hum Neurosci. 2021 Feb 3;15:621080. doi: 10.3389/fnhum.2021.621080 (PMC7886792; doi:10.3389/fnhum.2021.621080)
Supplement: Supplementary file 2 [file Table_2.docx]

**Supplementary Table 2:** T2DM therapeutic agents

| Therapeutic agent | Medication | Number of patients |
| --- | --- | --- |
| Dietary restriction |  | 11 |
| Insulin |  | 3 |
|  | Metformin | 11 |
|  | Metformin + sulfonylureas | 1 |
| Oral medication | Metformin + acarbose | 6 |
|  | Metformin + acarbose + sulfonylureas | 2 |
|  | Sulfonylureas | 1 |
|  | Acarbose | 1 |
|  | Metformin | 6 |
| Insulin+oral medication | Acarbose | 1 |
|  | Metformin + acarbose | 1 |
