# Supplementary material for: Functional Disconnection of the Angular Gyrus Related to Cognitive Impairment in Patients With Type 2 Diabetes Mellitus
Source: Front Hum Neurosci. 2021 Feb 3;15:621080. doi: 10.3389/fnhum.2021.621080 (PMC7886792; doi:10.3389/fnhum.2021.621080)
Supplement: Supplementary file 3 [file Table_3.docx]

**Supplementary Table 3:** The MoCA scores of the patients with type 2 diabetes mellitus (T2DM) and the healthy controls (HCs)

| Variable | T2DM (*n* = 44) | Controls  (*n* = 43) | | *P*-value |
| --- | --- | --- | --- | --- |
| Alternate connection | 1 (0,1) | 1 (0,1) | 0.07 | |
| Copy cube | 1 (0,1) | 1 (0,1) | <0.001 | |
| Draw clock | 2 (0,3) | 2 (1,3) | 0.71 | |
| Name | 3 (2,3) | 3 (3,3) | 0.05 | |
| Attention | 3 (2,3) | 3 (1,3) | 0.06 | |
| Calculation | 3 (1,3) | 3 (1,3) | 0.53 | |
| Language retelling | 2 (0,2) | 2 (0,2) | 0.01 | |
| Language fluency | 1 (0,1) | 1 (1,1) | 0.32 | |
| Abstract ability | 2 (0,2) | 2 (1,2) | 0.02 | |
| Delayed recall | 4 (0,4) | 4 (1,5) | 0.38 | |
| Orientation | 6 (3,6) | 6 (4,6) | 0.96 | |

Note: Variables that were not normally distributed are presented as their median (minimum, maximum), *P*-value for the Mann-Whitney *U*-test.
